# Draft Genome Analysis of *Christensenella minuta* DSM 22607, exhibiting an unusual expansion of transporter homologs of unknown function

**DOI:** 10.7150/jgen.43162

**Published:** 2020-02-21

**Authors:** David A Coil, Guillaume Jospin, Jonathan A. Eisen

**Affiliations:** 1 Genome Center, University of California, Davis, CA, United States.; 2 Department of Evolution and Ecology, University of California, Davis, CA, United States.; 3 Center for Population Biology, University of California, Davis, California, United States.; 4 Department of Medical Microbiology and Immunology, University of California, Davis, CA, United States.

**Keywords:** *Christensenella minuta*, * C. minuta* DSM 22607, Draft Genome Analysis

## Abstract

*Christensenella minuta* was first formally described in 2012 as a member of a novel species, genus, and proposed family of Christensenellaceae. *C. minuta* was later shown in one study to be part of the most heritable taxonomic group in the human gut microbiome and to be enriched in people with low body mass index (BMI). Mouse work demonstrated that injection of cultured *C. minuta* into germ-free mice prevented the onset of obesity after a fecal transplant to the mice from high BMI individuals. Here we describe the genome sequence of *C. minuta* DSM 22607. Examination and analysis of the annotation revealed an unusually high number of genes predicted to be involved in carbohydrate metabolism, many of which were multiple homologs of RbsA, RbsB and RbsC, which together make up the Ribose ABC Transport System. These genes may be also involved in quorum sensing which could potentially relate to the importance of *C. minuta* in the gut microbiome.

## Introduction

A strain of *Christensenella minuta*, isolated from human feces, was described in 2012 as a member of a novel species, genus, and proposed family of Christensenellaceae 1. *C. minuta* and the Christensenellaceae were highlighted as potentially of significant interest in 2014 in a paper involving studies of human twins and also experiments in mice 2. Findings from this paper include (a) that the Christensenellaceae family was the most heritable taxonomic group in the human gut microbiome, (b) that the Christensenellaceae and some other associated taxa were enriched in people with low body mass index (BMI) and (c) that injection of cultured *C. minuta* into germ-free mice prevented the onset of obesity after a fecal transplant to the mice from high BMI individuals. The mechanisms underlying these findings were not revealed in this study, and the story was further complicated by subsequent work demonstrating the pathogenic potential of *C. minuta* 3. In the last few years, there have been a number of studies showing human health associations with Christensenellacaeae, ranging from serum lipid levels to longevity to metabolic disorders 4. Based on the results of the 2014 paper and given the putative importance of *C. minuta* in human health, we sought to further examine *C. minuta* by sequencing its genome since none was publically available at the time. The genome of the type strain DSM 22607 has been since sequenced several times but the only published genome report was a data announcement 5. We report here sequencing efforts and a brief analysis of the genome of *C. minuta* strain DSM 22607. Specifically, we highlight the phylogenetically recent massive expansion of a set of transporter genes of unclear function.

## Methods

### Genome Sequencing

DNA from *C. minuta* DSM 22607 was obtained from the Deutsche Sammlung von Mikroorganismen und Zellkulturen (DSMZ). A paired-end library for Illumina sequencing was created using a Nextera XT Library Preparation Kit (Illumina). The library was size-selected (600-900 bp) on a Pippin Prep system (Sage Science) and sequenced on a PE300 bp run of an Illumina MiSeq at the UC Davis DNA Technologies Core Facility. Quality trimming, error correction, and assembly were performed by the A5-miseq assembly pipeline 6. Genome annotation was examined using RAST and the associated SEED Viewer 7.

### Whole genome tree and HMM scanning

Genomes of all 9499 sequenced type strains were downloaded from NCBI on August 23rd 2019. These were all run through Phylosift's 8 search (lastal 9) and align (hmmalign 10) functions to extract 37 mostly single copy marker genes used to build a large “whole genome” phylogenetic tree (FastTreeMP, 11). From *C. minuta*'s location on the tree, we walked back up to an internal node to include two large clades around *C. minuta* DSM 22607. *Streptococcus porcinus* was selected as belonging to a closely related clade to the three described previously. This resulted in 373 taxa being selected for a maximum likelihood tree using RAxML 8.2.11 12 and the PROTGAMMABLOSUM62 substitution matrix, and the following parameters (-p 8 -x 47 -T 48 -f a -N 1000). Using the *C. minuta* DSM 22607 genome annotation from RAST, we extracted the sequences of all RbsA (n=42), RbsB (n=20), and RbsC (n=41) proteins. Each of these sequences was then compared to the progidal2.6.3 13 predicted proteins from the 9,499 genomes using BLAST2.9 14. Predicted proteins matching the reference proteins below an e-value of 1e-30, were counted and summed for each genome and added to the tree's tip labels. Any hits below an e-value of 1e-30 were counted for each marker. These numbers were added to the tree's tip labels.

### Average nucleotide identity (ANI)

We selected all 11 publicly available *Christensenella* genomes and compared their average nucleotide identity using fastANI 15 with default settings (fragment size of 3000bp).

## Results & Discussion

### Assembly/Validation

In total 770,216 Illumina reads were used in the assembly, producing a draft genome of 2,942,834 bp, in 42 contigs, with an N50 of 148,400 bp at a coverage of ~79X. Genome completeness was estimated using CheckM 16, which searches for single-copy, highly conserved markers, and it gave a completeness estimate of 98.4 % and contamination estimate of .81%.

### Annotation

We sought to make predictions regarding the unique metabolic characteristics of *C. minuta*, but this was complicated by the lack of genome sequences for close relatives. According to the original species description of *C. minuta*, the closest relatives were *Caldicoprobacter oshimai*, *Tindallia californiensis*, and *Clostridium ganghwense* 1 (which aren't even from the same family within Clostridiales). Both *C. oshimai* and *T. californiensis* have sequenced genomes, and these were used here for an initial comparison. The RAST Seed Viewer also highlights the genome that the RAST automated analysis identifies as having the most similar predicted metabolic profile within the collection of complete genomes in their database. In this case the genome is that of *Clostridium novyi*. Another organism used here for comparison was *Methanobrevibacter smithii* which co-occurs with *C. minuta*, has also been associated with lower BMI, but was not found to be highly heritable 2. Since *C. minuta* was described, two other *Christensenella* species have been proposed and their genomes have been sequenced (*C. timonensis* and *C. massiliensis*) 17,18. However, it is important to note that the descriptions of these two isolates were not done using accepted chemotaxonomic analyses and thus whether or not these should be considered true members of the *Christensenella* genus is unclear. Similarly, in the phylogenetic tree of all bacterial type strains (see below), *Catabacter hongkongensis* appears to group with the three sequenced Christensenella species, suggesting that further taxonomic revision in this group may be required.

In examination of the RAST annotation of the *C. minuta* DSM 22607 genome, the most striking characteristic to us was the large number of genes (n=409) predicted to be involved in carbohydrate utilization (Table 1). This is striking in that the total number is much higher than that seen for genomes of the close relatives we examined, with the exception of *C. timonensis* which has 434. In addition, the genome of the organisms flagged by RAST as having the most similar predicted metabolic profile, *C. novyi*, has much fewer (201) as does the genome of one of the key co-occurring organisms, *M. smithii*, which has only 90. There are many reasons why the two *Christensenella* species could have more carbohydrate metabolism genes annotated. One possibility is simply inaccuracies in annotation. But it could also be a reflection of true higher numbers of carbohydrate metabolizing genes in these species. We did not view these numbers of putative carbohydrate metabolism genes as being a precise measure of metabolism in these organisms but instead used this annotation as a means to focus our attention on these genes to see if any more light could be shed on possible unusual features of *Christensenella*.

### RbaA/B/C genes

Examination of the predicted carbohydrate metabolism genes in more detail revealed a more striking finding. A significant fraction of all these genes in Christensenella (127 in total) were annotated as encoding homologs of RbsA, RbsB or RbsC which together make up the Ribose ABC Transport System. Early work focused on the importance of these genes in the transport of D-ribose 19,20, but there is more recent evidence that homologs of these genes may be involved in quorum sensing instead of ribose transport 21-24. This is perhaps not surprising given the fact that the quorum sensing AI-2 autoinducer is derived from the ribosyl moiety of *S*-ribosylhomocysteine (a potential target of the transporters). Given the recently hypothesized role of AI-2 in structuring gut microbial communities 25 we hypothesize that the massive expansion of the Rbs transporters in this lineage may relate to quorum-sensing. Furthermore, this could relate to the demonstrated importance of this organism in structuring the gut microbial community. In particular, it could be that members of the *Christensenella* group act as “foundation” species in the human gut and that they directly structure the microbial community through quorum sensing mediated interactions.

**Figure 1:**
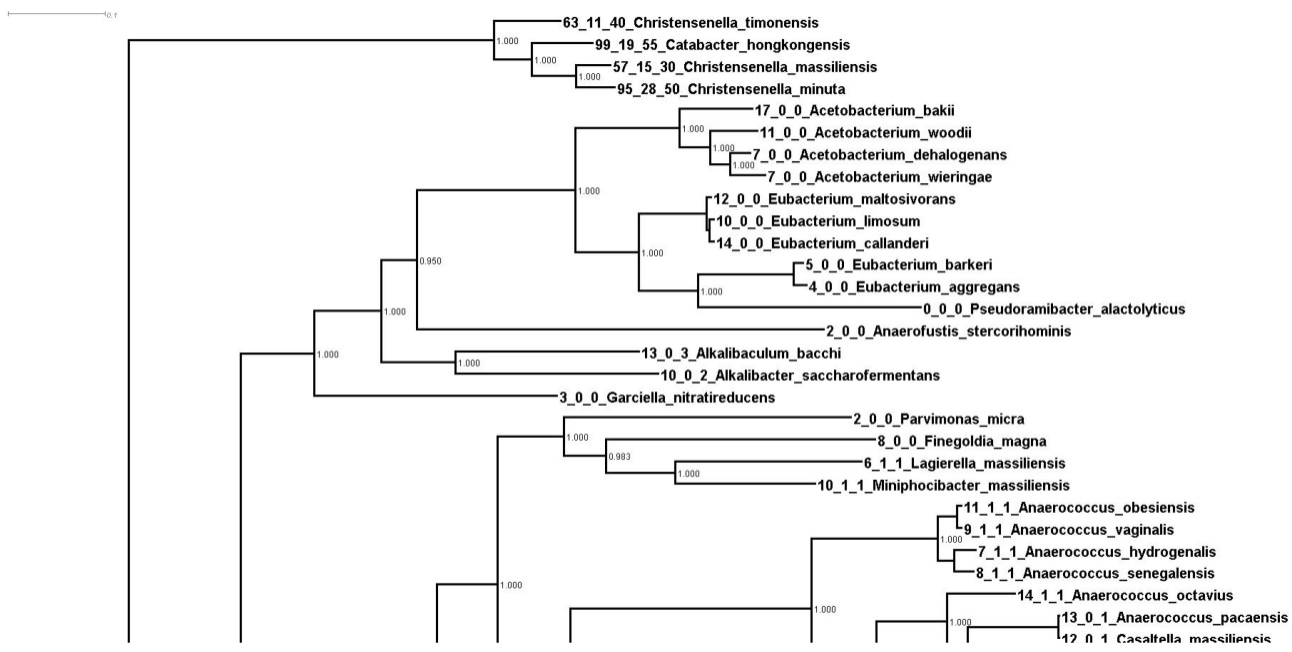
** Phylogenetic relationships of Christensenella based on whole genome analysis**. Shown is a zoom in of a larger phylogenetic tree of 9499 type strains generated from concatenated marker genes using Phylosift. Values at the node represent bootstrap values from RaxML.

In order to understand the expansion of these genes, we started by constructing a “whole genome tree” of all the available bacterial type strains at NCBI. We downloaded 9,499 type strain genomes and ran them through Phylosift in order to build a concatenated marker tree. Once the tree was constructed, we overlaid the number of RbsA, RbsB, and RbsC proteins onto the tips of the tree. A portion of this tree, containing the 372 strains phylogenetically closest to *Christensenella* (plus an outgroup, *Streptococcus porcinus*) is shown in Supplemental Figure 1. The portion of the tree containing the *Christensenella* clade is shown in Figure 1.

In Figure 1 we can see firstly that the *Christensenella* clade is not particularly phylogenetically close to the other bacteria in this tree. In fact the group is an outgroup to all other 368 strains in this tree (Supplemental Figure 1). Additionally, most of the bacteria in this tree encode relatively few RbsA/B/C homologs. There are multiple possible explanations for the pattern seen in number of these genes. These include lateral gene transfer into or duplication events in the ancestry of species with more of these genes, or alternatively, ancestral events such as these and then loss in lineages with fewer numbers. We consider it most likely that there was an expansion (either by duplication or lateral gene transfer) of these genes within the *Christensenella* clade (and a few other lineages).

As another way of looking at this data, we performed a BLAST-p 26 search (e-value cutoff of 1e ^-30^) using all 127 RbsA/B/C sequences from *C. minuta* DSM22607 against all 9,499 downloaded type strain genomes. The 10 organisms with the most RbsA/B/C predicted proteins matching at an e-value of 1e^-30^ or better in their genomes from this search are listed in Table 2, along with *C. timonensis* and *C. massiliensis* (duplicate genomes from the same strains were removed). While there are not many homologs of any of the RbsA/B/C proteins present in the close relatives to *C. minuta*, there are a few other distantly related bacteria (Rhizobiales) with even more homologs. However, the possession of a large number of RbsB gene copies appears unique to the *Christensenella* lineage. When all 9,499 genomes are ranked by the number of RbsB homologs alone, the top 10 species are all *Christensenella*, *Catabacter*, and one *Clostridium bolteae* isolate (data not shown).

Since we submitted the genome sequence of *C. minuta* DSM 22607 to NCBI in 2016, the same strain (DSMZ 22607) has had its genome sequence deposited several more times. In order to ensure that the results reported here were not due to sequencing or assembly artifacts we compared all these DSMZ 22607 assemblies themselves as well as the RAST annotations of the genes of interest. The average nucleotide identity (ANI) between assemblies of this strain was >99.98% demonstrating that they are virtually identical. We found the same with the RAST results, where the number of annotated Rbs transporters was between 126-128 per genome in every case.

Given the potential importance (positive and negative) of *C. minuta* in human health, and the fact that interventions based on this bacteria are already being considered, genomic analysis paired with functional experiments will be critical for a deeper understanding of this organism. In particular, the apparent expansion of genes encoding RbsA/B/C in this lineage warrants investigation.

### Nucleotide sequence accession numbers

This Whole Genome Shotgun project has been deposited at DDBJ/ENA/GenBank under the accession LWGY00000000. The version described in this paper is version LWGY01000000.

## Supplementary Material

Supplemental Figure 1.

## Figures and Tables

**Table 1:** ***C. minuta* and selected organisms for comparison.** Shown are the results from RAST annotation of these genomes.

Isolate	Total Carbs	Monosaccharides	D-ribose	Accession
*C. timonensis*_Marseille_P2437	434	125	91	GCF_900087015.1
*C. minuta*_DSM_22607_UCD	409	179	127	GCA_001652705.1
*C.* sp_AF73_05CM02	405	192	140	GCF_001678845.1
*C. massiliensis*_Marseille_P2438	300	110	77	LT700187.1
*C.* sp_Marseille_P3954	228	88	46	GCF_900604345.1
*Caldicoprobacter oshimari*	218	74	7	GCA_000526435.1
*Clostridium novyi*	201	41	7	GCF_000014125.1
*Tindallia californiensis*	169	18	13	GCA_900107405.1
*Methanobrevibacter smithii*	90	5	1	GCF_000016525.1

**Table 2:** The 10 organisms with the most RbsA/B/C predicted proteins using our approach

Rank	Genome	RbsA	RbsB	RbsC	Total	
1	*Kaistia_algarum*	140	6	85	231	GCF_002930635.1
2	*Mesorhizobium_helmanticense*	130	7	66	203	GCA_003034915.1
3	*Mesorhizobium_sanjuanii*	125	7	62	194	GCF_002529485.1
4	*Christensenella_sp.*_AF73-05CM02	104	27	55	186	GCF_002529485.1
5	*Catabacter_hongkongensis_strain*_HKU16	99	19	55	173	GCA_000981035.1
6	*Christensenella_minuta_strain*_DSM_22607	95	28	50	173	GCA_001652705.1
7	*Rhizobium_album*	111	4	57	172	GCF_003122325.1
8	*Bauldia_litoralis*	102	4	60	166	GCF_900104485.1
9	*Mesorhizobium_ephedrae*	107	6	46	159	GCF_003012745.1
10	*Kaistia_granuli*	100	4	54	158	GCF_000380505.1
49	*Christensenella_timonensis*	63	11	40	114	GCF_900087015.1
71	*Christensenella_massiliensis*	57	15	30	102	GCA_900155415.1
