# Supplementary material for: Draft Genome Analysis of Christensenella minuta DSM 22607, exhibiting an unusual expansion of transporter homologs of unknown function
Source: J Genomics. 2020 Feb 21;8:25–9. doi: 10.7150/jgen.43162 (PMC7064737; doi:10.7150/jgen.43162)

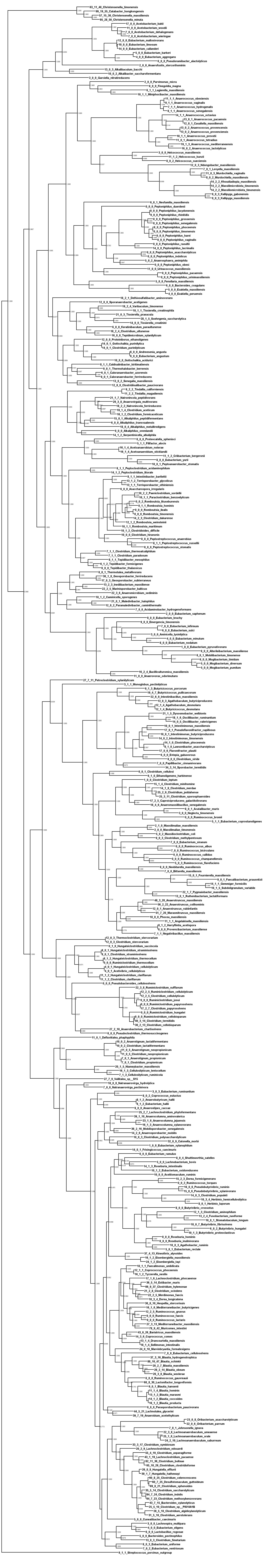

Supplement: Supplementary file 1 — Supplemental Figure 1. [file jgenv08p0025s1.pdf]
